# The effects of the first versus second glaucoma drainage implant surgery in patients with primary open-angle glaucoma

**DOI:** 10.1186/s12886-023-03247-y

**Published:** 2023-12-14

**Authors:** Shuu Morita, Teruhiko Hamanaka, Tetsuro Sakurai, Satoshi Watanabe, Yoshihito Sakanishi, Nobuo Ishida, Nobuyuki Ebihara

**Affiliations:** 1 Department of Ophthalmology, Juntendo University Urayasu Hospital, Chiba, Japan; 2 Department of Ophthalmology, Japanese Red Cross Medical Center, 4-1-22, Hiroo, Shibuya-ku, Tokyo, 156-8935 Japan; 3 School of General and Management Studies, Suwa University of Science, Nagano, Japan; 4 Ishida Eye Clinic, Niigata, Japan

**Keywords:** The first/second glaucoma-drainage-implant, Primary open-angle glaucoma

## Abstract

**Background:**

To investigate the outcome of non-valved glaucoma drainage implant surgery (GDIS) in primary open-angle glaucoma (POAG) patients divided in the first GDI group (patients who underwent the first GDIS) and the second GDI group (patients who underwent the second GDIS because of the failed first GDIS).

**Methods:**

Intraocular pressure (IOP), visual acuity (VA), visual field defect (VFD), medication score (MS), survival rate of GDIS, complications, and patient background was retrospectively analyzed. Two success criteria were set: Criteria (1) IOP reduction ≥ 20% and 5 < IOP ≤ 21, Criteria (2) IOP reduction ≥ 20% and 5 < IOP ≤ 14.

**Results:**

There were 136 eyes of 109 patients in the first GDI group and 32 eyes of 27 patients in the second GDI group. In the first GDI group and II, mean preoperative IOP was 26.7 ± 6.7 mmHg and 23.7 ± 3.5 mmHg, respectively (*P* = 0.09). No statistically significant difference in postoperative IOP reduction was found between the two groups (*P* = 0.39). At 5-years postoperative, the Criteria 1 (Criteria 2) survival rate in the first GDI group and the second GDI group was 60.4% (31.7%) and 61.2% (25.6%), respectively (Criteria 1: hazard ratio [HR]: 0.64, 95% confidence interval [CI]: 0.30–1.35 [*P* = 0.24]; Criteria 2: HR: 0.81, 95% CI: 0.46–1.44, *P* = 0.48). No significant difference in VA, VFD change, MS, or complications was observed. Young patient age was the only significant factor for failure in the first GDI group (odds ratio: 0.95, 95% confidence interval: 0.91-1.00, *P* = 0.03).

**Conclusion:**

The second GDIS may be as effective as the first GDIS for IOP reduction in POAG patients, however, there is a high risk of failure in young-age patients and the surgery may be ineffective in eyes requiring Criteria 2.

## Background

Although glaucoma drainage implant surgery (GDIS) has been widely used for the treatment of glaucoma, the postoperative outcome is known to differ depending on the type of glaucoma being treated. It has been reported that the nature of secondary glaucoma, such as neovascular, uveitic, congenital, or pediatric glaucoma, may be refractory to intraocular pressure (IOP) control even when GDIS is performed [1–4]. The outcome of glaucoma surgery including the use of a glaucoma drainage implant (GDI) in cases of neovascular glaucoma or uveitic glaucoma may be strongly affected by multiple factors. The idea of implanting a second tube if the first GDI fails may come from the findings in a representative study proving that lower mean IOP can be achieved with larger implant plate sizes [5]. Therefore, it may be interesting to compare the first and second GDIS by restricting the investigation to only POAG patients. Most previous studies on the first (primary) or second GDIS have involved different types of glaucoma, including refractory glaucoma. However, and to the best of our knowledge, there has been only one published report on the efficacy of GDIS in eyes with simple glaucoma, and that study included both primary open-angle glaucoma (POAG) and exfoliation glaucoma cases [6].

In POAG, unlike neovascular or uveitic glaucoma, the reduction of IOP post surgery is the primary factor used to evaluate the efficacy of a GDI. Thus, we theorized that it may be important to investigate the efficacy of non-valved GDIS by limiting the eyes investigated to only those with POAG.

The purpose of this present study was to retrospectively investigate the outcomes post first and secondary GDIS using a non-valved GDI (i.e., the double-plate Molteno and Baerveldt glaucoma implants (BGI) 250mm^2^ and BGI 350mm^2^) by limiting the eyes to cases afflicted with POAG.

## Methods

The protocols of this retrospective, non-randomized, medical-record-review study were approved by the Institutional Review Board of the Japanese Red Cross Medical Center, Tokyo, Japan, and in accordance with tenets set forth in the Declaration of Helsinki, written informed consent was obtained from all subjects prior to the use of their medical records. This retrospective study involved 168 eyes of 136 consecutive Japanese POAG patients who underwent GDIS by a single surgeon (T.H.) at the Japanese Red Cross Center. In this study, the inclusion criteria was POAG and juvenile open angle glaucoma (JOAG) patients seen between April 1998 and April 2021 who were follow-up period of for a minimum of more than 6-months postoperative. The medical-record data of those 136 patients was retrospectively reviewed after dividing the patients into the following two groups: the first GDI group (eyes that had undergone the first GDIS [136 eyes of 109 patients]), and the second GDI group (eyes that had undergone the second GDIS after the first GDIS failed [32 eyes of 27 patients]). In all treated eyes, a non-valved GDI (i.e., a double-plate Molteno, BGI 250mm^2^, or BGI 350mm^2^) was used, and no glaucoma surgery was performed between the first and the second GDIS. The first implanted GDIs were mostly placed in the inferior temporal quadrants, while and the second GDIs that were implanted were inserted in opposite quadrants. All eyes with minimum of 6 months follow-up post implantation of the GDI were included in the study. In the previous study, we defined refractory glaucoma as the eyes having a high IOP of more than 40 mmHg [7] or 35 mmHg [8] and/or a severe visual field defect (VFD) (i.e., a visual field (VF) deterioration of ≥ Stage V in the Aulhorn-Greve classification) [7, 8]. In this present study, the eyes that had an IOP of ≥ 35 mmHg and/or a severe VF deterioration of ≥ Stage V (Aulhorn-Greve classification) received the combined surgery of Trabeculectomy (TRAB) and GDI (TRAB/GDI). A successful surgical outcome was defined by the following two criteria: Criteria 1: an IOP of ≤ 21 mmHg and an IOP reduction of ≥ 20%, and Criteria 2: an IOP of ≤ 14 mmHg and an IOP reduction of ≥ 20%. If the first GDIS failed due to Criteria 1, the second GDIS was performed. It should be noted that the reason for including Criteria 2 is that an IOP of ≤ 14 mmHg is important for preventing any further progression of VFD in eyes afflicted with the advanced stage of POAG [9–12]. The eyes with an IOP of ≤ 5 mmHg, an IOP of > 21 mmHg at two consecutive follow-up visits, and no light perception at the post-operative visit were regarded as failure. In the eyes that underwent corneal transplantation or pars plana vitrectomy (PPV), the observation period was considered finished at the date of the corneal transplantation or PPV.

The following background data of the eyes pre GDIS was reviewed: (1) patient age at the time of undergoing GDIS, (2) patient age at the time of the POAG diagnosis before or after the age of 40 years, (3) a family history of POAG within the first generation, (4) gender, (5) the operated eye, (6) previous glaucoma surgeries performed (i.e., trabeculotomy [TLO] and TRAB), (7) lens status (phakic or pseudophakic), (8) preoperative (pre-op) IOP (i.e., the maximum IOP within 42 days prior to surgery), (9) pre-op glaucoma medication score within 42 days (joint instillation was counted as two medications, and one acetazolamide oral tablet was counted as one medication), (10) pre-op best-corrected visual acuity (BCVA, Snellen), (11) pre-op VF deterioration evaluated by Aulhorn-Greve classification, (12) type of GDI used, (13) location of the plate, and (14) ligation site (ligated by 7 − 0 nylon suture and Ethicon 8 − 0 Vicryl Suture [Johnson & Johnson, New Brunswick, NJ] in the anterior chamber and on the sclera, respectively). The following postoperative data was also reviewed: data on IOP, glaucoma medication score, and complications, including corneal endothelial cell (CEC) density loss, collected at the follow-up examinations performed at 1 month, 3 months, 6 months, 1 year, each subsequent year, and the final visit post surgery. Moreover, postoperative BCVA and VF data were collected at the final follow-up visit.

The parameters of IOP, glaucoma medication score, BCVA, VF, complications, and Caplan-Meier survival curve were compared between the first GDI group and the second GDI group, including or excluding the eyes that underwent combined TRAB/GDI. In addition, multivariate and univariate analysis was used to investigate the influence of the patients’ background (i.e., parameters 1–14, listed above) on the outcome of the surgeries in each group.

The detailed procedures of the GDIS performed were as described elsewhere [7]. Briefly, all tubes in the combined TRAB/GDIS eyes or some eyes in which only GDIS was performed were ligated in the anterior chamber, while the tubes in the remaining eyes in which only GDIS was performed were ligated on the sclera. Two or three Sherwood slits (venting slits) were made between the limbus and the ligation on the sclera. In the eyes with a ligated tube in the anterior chamber (i.e., all combined TRAB/GDIS cases and some of the cases in which only GDIS was performed), the ligation of the tube was released via the use of an argon laser at around 5-weeks postoperative if the IOP was raised to 21 mmHg (GDIS only cases), or if it reached the high teens (combined TRAB/GDIS cases). In the TRAB/GDIS cases, after completion of GDIS, TRAB with mitomycin C was performed at the 3- or 9-o’clock position next to the site in which the tube was inserted. Laser suture lysis of the flap was performed when the IOP started to elevate more than 15 mmHg.

### Statistical analysis

For categorical variables data were summarized by frequency (%) and compared between the two groups using the Fisher exact test or the Chi-square test. All continuous data were evaluated using the Mann-Whitney U test.

IOP changes post GDIS were evaluated by Friedman’s test for multiple comparisons, followed by the Wilcoxon signed rank test in each group. The Mann-Whitney U test was used to evaluate whether or not there was a significant difference in IOP change and IOP reduction rate between the two groups. Changes of glaucoma medications in each group were also evaluated via the same methods.

Cumulative success rate was calculated by Kaplan-Meier survival curve analysis, and the comparison of the cumulative success rate between those groups was evaluated by use of the log-rank test followed by examination with the Cox proportional hazard model. Risk factors for failure were extracted among the above-listed 14 parameters using logistic regression analysis. Risk factors with a *P*-value of < 0.1 in univariate analysis were included in the multivariate analysis. A *P*-value of < 0.05 was considered statistically significant.

All statistical analyses were performed using R version 4.0.2 software for Windows (R Foundation for Statistical Computing, Vienna, Austria).

## Results

The mean postoperative follow-up period in the first and second GDI groups was 60.0 ± 41.6 months and 53.0 ± 27.7 months, respectively. The patient demographics are outlined in Table 1. In this study, 136 eyes of 109 patients (mean age: 61.0 ± 11.2 years) in the first GDI group and 32 eyes of 27 patients (mean age: 60.5 ± 11.4 years) in the second GDI group were investigated. In each group, there were more male than female patients, and number of males in the second GDI group was significantly more than that in the first GDI group (*P = 0.01*). There was no significant difference between the number of eyes with and without a history of previously undergoing cataract surgery, but the numbers of previous TRAB surgeries received in the eyes of the second GDI group were significantly more than those in the first GDI group (*P = 0.01*). The mean pre-op IOP and glaucoma medication score was 26.7 ± 6.7 mmHg and 3.9 ± 1.4, respectively, in the first GDI group, and 23.7 ± 3.5 mmHg and 3.9 ± 1.1, respectively, in the second GDI group. Between the first GDI group and the second GDI group, there was no significant difference in pre-op IOP (*P* = 0.09) and glaucoma medication score (*P* = 0.68), and there was also no significant difference in family history of glaucoma or the type of GDI used. The first GDIs were mostly inserted in the inferior temporal quadrants (88.5%), while the second GDIs were inserted in the upper temporal (56.3%) or upper nasal quadrants (31.3%) (Table 1). There was no significant difference in IOP reduction range/rates between the first GDI group (9.1 ± 8.3 mmHg/31.3 ± 28.2%) and the second GDI group (7.0 ± 5.2 mmHg/24.0 ± 23.5%) (*P* = 0.39/0.66) (Table 2). Even when the eyes with combined TRAB/GDI were excluded, there was no difference between the first GDI group and the second GDI group in regard to IOP reduction range/rates and reduction range/rate of glaucoma medications used (IOP reduction range/rates: *P* = 0.30/0.35; glaucoma medication reduction range/rate: *P* = 0.18/0.37) (Table 2).

**Table 1:** Patient demographics and baseline ocular characteristics

	the first GDI group	the second GDI group	*P-*value
No. of eyes (patients)	136 (109)	32 (27)	
Age at surgery (mean ± SD years)	61.0 ± 11.2	60.5 ± 11.4	1.00
Age at diagnosis: <40 years:〈n, (%)〉	36 (27.7%)	12 (41.4%)	0.22
Family history: yes:〈n, (%)〉	46 (34.3%)	13 (40.6%)	0.64
Gender: male〈n, (%)〉	98 (72.1%)	30 (93.8%)	**0.01**
Operated eye: right〈n, (%)〉	64 (47.1%)	13 (40.6%)	0.64
Previous glaucoma surgeries			
TLO (mean ± SD)	0.2 ± 0.6	0.2 ± 0.5	0.65
TRAB (mean ± SD)	1.1 ± 1.0	1.6 ± 1.2	**0.01**
Lens status: phakic〈n, (%)〉	50 (37.0%)	12 (37.5%)	1.00
Preoperative data			
IOP (mmHg) (mean ± SD)	26.7 ± 6.7	23.7 ± 3.5	0.09
Glaucoma medication score (mean ± SD)	3.9 ± 1.4	3.9 ± 1.1	0.68
BCVA (mean ± SD)	0.7 ± 0.4	0.6 ± 0.5	0.55
VF (mean ± SD)	4.0 ± 1.5	4.2 ± 1.5	0.57
Type of GDI〈n, (%)〉			0.22
BGI250	49 (36.0%)	16 (50.0%)	
BGI350	71 (52.2%)	15 (46.9%)	
Double-Plate Molteno Implant	16 (11.8%)	1 (3.1%)	
Location of the plate〈n, (%)〉			**< 0.01**
Temporal upper	7 (5.7%)	18 (56.3%)	
Temporal lower	108 (88.5%)	3 (9.4%)	
Nasal lower	5 (4.1%)	1 (3.1%)	
Nasal upper	2 (1.6%)	10 (31.3%)	
Ligation site: in the anterior chamber〈n, (%)〉	72 (55.3%)	17(53.1%)	0.97

Mann-Whitney U test

Fisher’s exact test / chi-square test

BCVA, best-corrected visual acuity; BGI, Baerveldt glaucoma implant; GDI, glaucoma drainage implant; IOP, intraocular pressure; SD, standard deviation; TLO, trabeculotomy; TRAB, trabeculectomy; VF: visual field

**Table 2:** Comparison of IOP and medication score before GDIS and at final visit, and reduction in the first GDI group and the second GDI group

IOP				Medication Score			
	the first GDI group	the second GDI group	***P*** **-value**		the first GDI group	the second GDI group	***P*** **-value**
**All Cases**							
No. of eyes	136	32		No. of eyes	131	32	
IOP reduction range (mmHg) (mean ± SD)	9.1 ± 8.3	7.0 ± 5.2	0.39	The reduction range (mmHg) (mean ± SD)	0.8 ± 2.0	0.7 ± 2.0	0.55
IOP reduction rate (%) (mean ± SD)	31.3 ± 28.2	24.0 ± 23.5	0.66	The reduction rate (%) (mean ± SD)	11 ± 59	15 ± 47	1.00
**Excluding TRAB Cases**							
No. of eyes	96	23		No. of eyes	96	23	
IOP reduction range (mmHg) (mean ± SD)	8.2 ± 7.5	5.9 ± 5.2	0.30	The reduction range (mmHg) (mean ± SD)	0.7 ± 1.8	0.3 ± 2.0	0.18
IOP reduction rate (%) (mean ± SD)	29.3 ± 22.0	23.1 ± 23.5	0.35	The reduction rate (%) (mean ± SD)	10 ± 56	3 ± 44	0.37

Mann-Whitney U test

There was no significant difference of IOP reduction range/rates between the first GDI group and the second GDI group

There was no significant difference in the reduction range/rate of glaucoma medications between the first GDI group and the second GDI group

GDIS, glaucoma device implant surgery; IOP, intraocular pressure; SD, standard deviation; TRAB, trabeculectomy

**Fig. 1:**
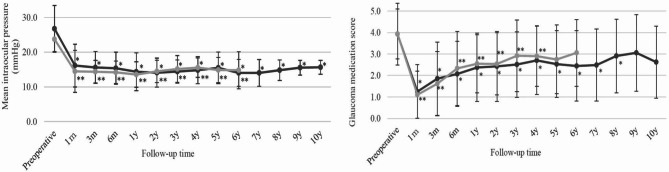
Intraocular pressure (IOP) and medication score prior to surgery and during the postoperative follow-up period in Groups I and II. Friedman’s test: *P* < 0.01. IOP reduction was statistically significant at each time point. ^*^P < 0.05 vs. preoperative for the first GDI group, ^**^P < 0.05 vs. preoperative for the second GDI group

**Fig. 2:**
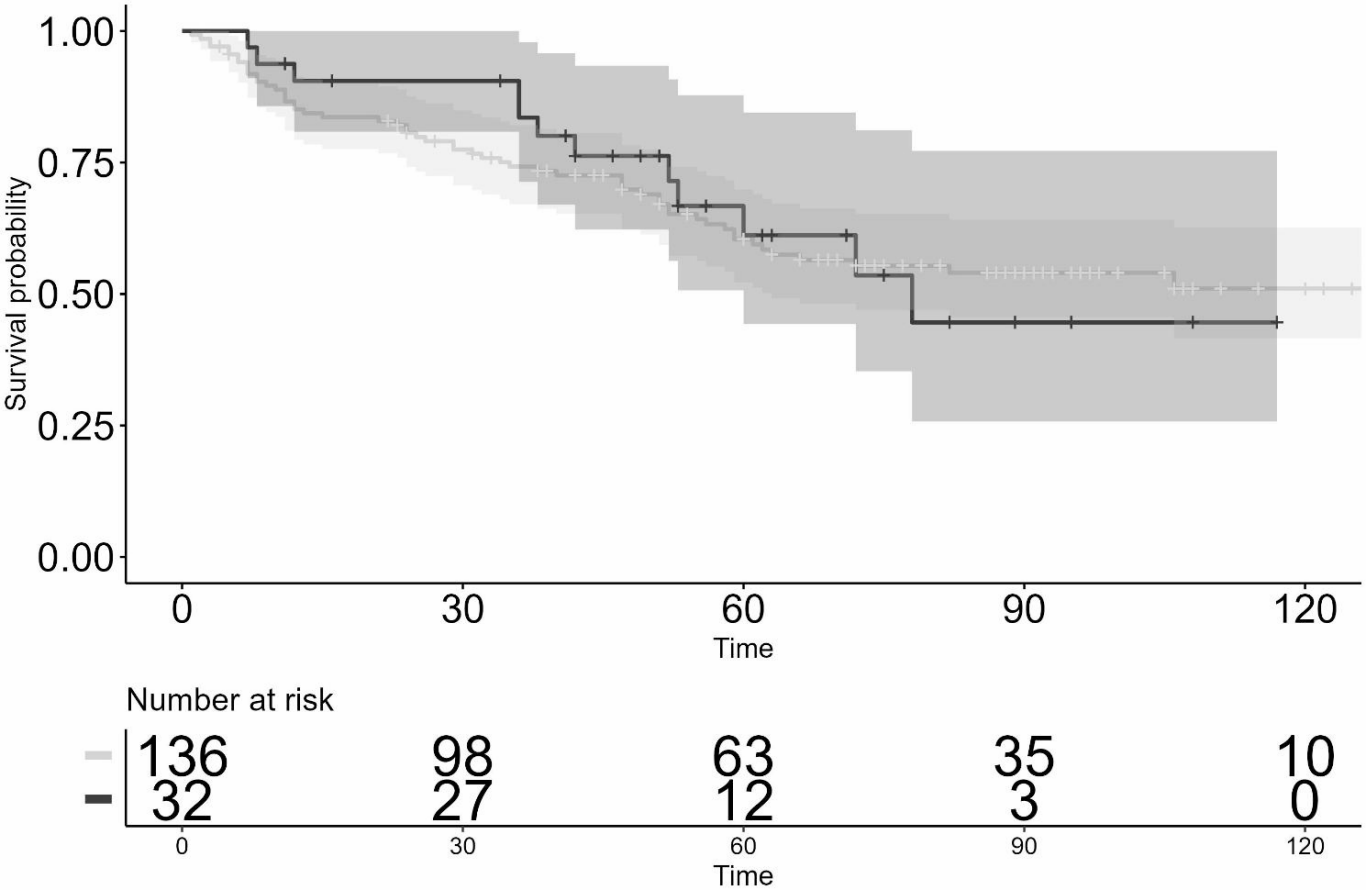
Kaplan-Meier survival curves evaluated by Criterion 1: 5 < IOP ≤ 21 mmHg. Log-rank test: *P* = 0.80, Cox proportional hazard model: Hazard ratio (HR): 0.64, 95% confidence interval (CI): 0.30–1.35, *P* = 0.24.　Shaded regions indicate the 95% confidence intervals (the first GDI group: thin shaded region, the second GDI group: thick shaded region). horizontal axis: months

**Fig. 3:**
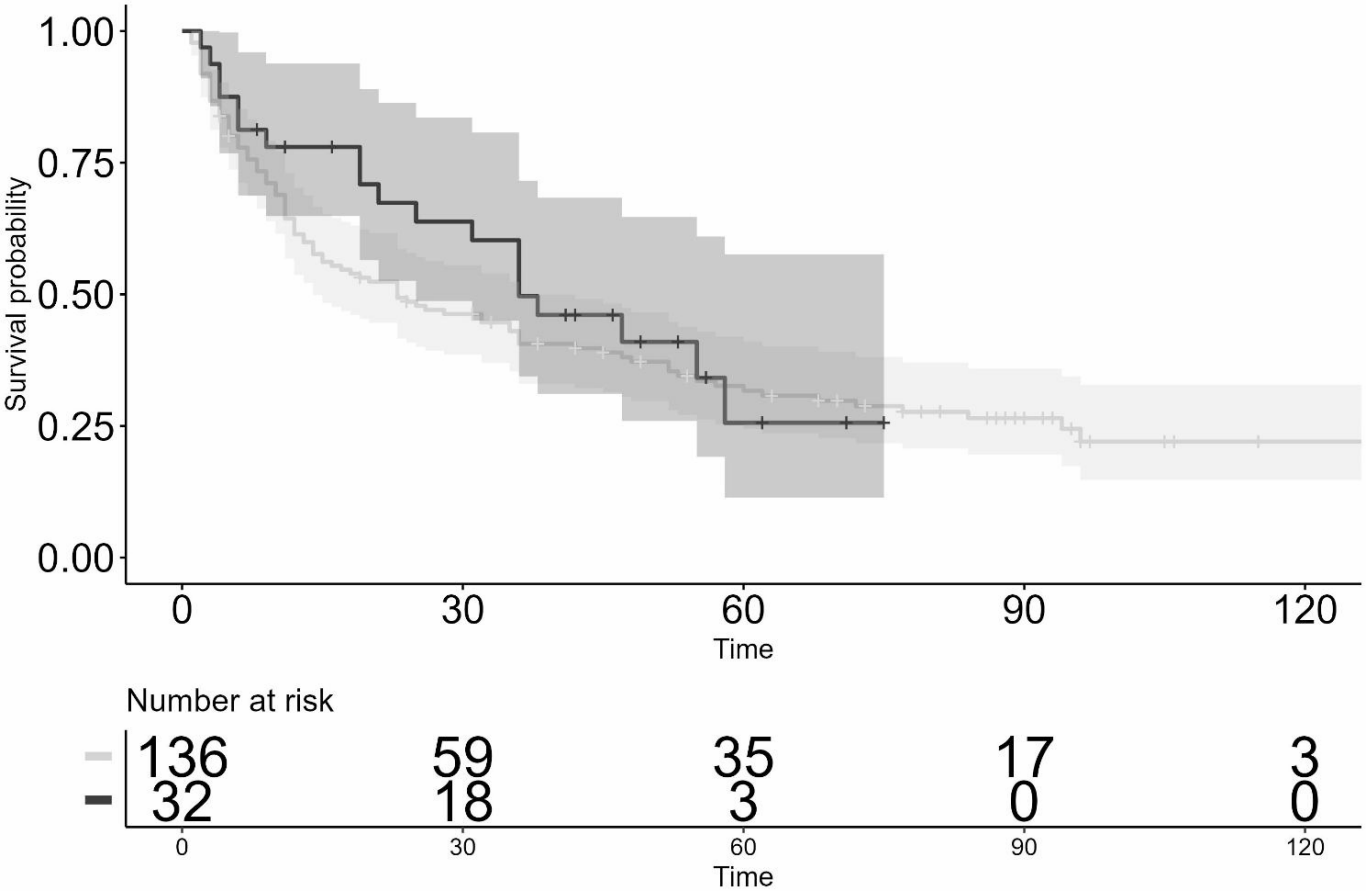
Kaplan-Meier survival curves evaluated by Criterion 2: 5 < IOP ≤ 14mmHg. Log-rank test: *P* = 0.40, Cox proportional hazard model: HR: 0.81, 95% CI: 0.46–1.44, *P* = 0.48. Shaded regions indicate the 95% confidence intervals (the first GDI group: thin shaded region, the second GDI group: thick shaded region). horizontal axis: months

As for the IOP lowering effect, post surgery, the mean pre-op IOP was significantly reduced from 26.7 ± 6.7 mmHg to 15.6 ± 2.0 mmHg for 10 years in the first GDI group (*P* < 0.05) and from 23.7 ± 3.5 mmHg to 14.8 ± 5.4 mmHg for 6 years in the second GDI group (*P* < 0.05) (Fig. 1). Moreover, the mean number of glaucoma medications used was significantly reduced from 3.9 ± 1.4 to 2.9 ± 1.7 at 8 years postoperative in the first GDI group (*P* < 0.05), and from 3.9 ± 1.1 to 2.9 ± 1.4 at 4 years postoperative in the second GDI group (*P* < 0.05) (Fig. 1). The Kaplan-Meier survival-curve rate as defined with Criteria 1 (5 < IOP ≤ 21 mmHg) at 3-, 5-, and 10-years postoperative in the first GDI group was 74.2%, 60.6%, and 51.0%, respectively, and at 3- and 5-years postoperative in the second GDI group was 83.6% and 61.2%, respectively. There was no statistically significant difference in the survival-curve rates between the first GDI group and the second GDI group (*P* = 0.80) (Fig. 2). The survival-curve rates corrected by patient background with the Cox proportional hazard model also showed the same results (hazard ratio [HR]: 0.64, 95% confidence interval [CI]: 0.30–1.35 [*P* = 0.24]). On the other hand, the Kaplan-Meier survival-curve rate as defined via Criteria 2 (5 < IOP ≤ 14 mmHg) at 3-, 5-, and 10-years postoperative in the first GDI group was 40.6%, 31.7%, and 22.0%, respectively, and at 3- and 5-years postoperative in the second GDI group was 49.6% and 25.6%, respectively. Hence, there was no statistically significant difference in the survival-curve rates between the first GDI group and the second GDI group (*P* = 0.40) (Fig. 3). The survival rates corrected by patient background with the Cox proportional hazard model were also found to be the same (HR: 0.81, 95% CI: 0.46–1.44, *P* = 0.48).

**Fig. 4:**
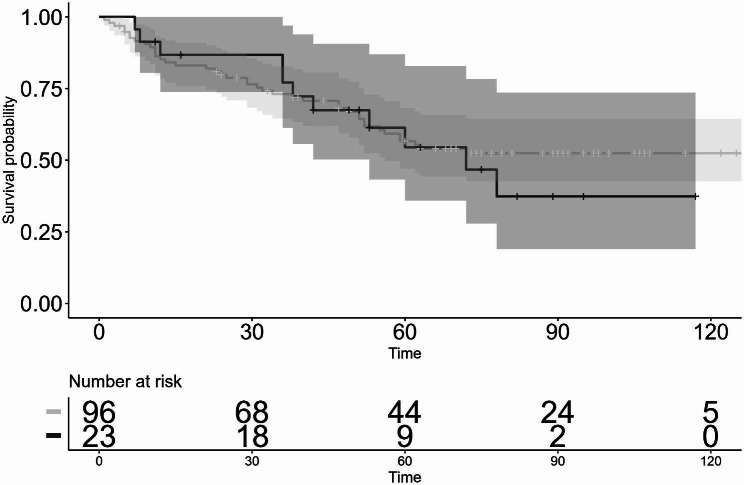
Kaplan-Meier survival curves, excluding the eyes that underwent combined TRAB and GDIS, evaluated by Criterion 1: 5 < IOP ≤ 21 mmHg. GDIS, glaucoma device implant surgery; IOP, intraocular pressure; TRAB, trabeculectomy. Log-rank test: *P* = 0.2, Cox proportional hazard model: HR: 0.81, 95% CI: 0.46–1.44, *P* = 0.48. Shaded regions indicate the 95% confidence intervals (the first GDI group: thin shaded region, the second GDI group: thick shaded region). horizontal axis: months

**Fig. 5:**
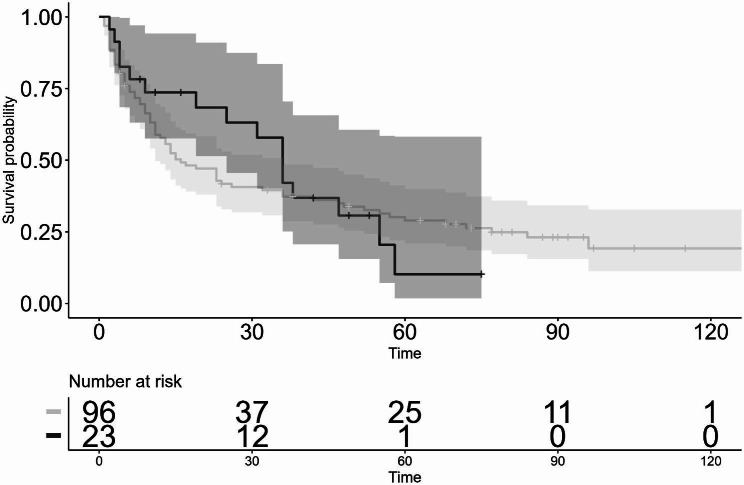
Kaplan-Meier survival curves, excluding the eyes that underwent combined TRAB and GDIS, evaluated by Criterion 2: 5 < IOP ≤ 14 mmHg. GDIS, glaucoma device implant surgery; IOP, intraocular pressure; TRAB, trabeculectomy. Log-rank test: *P* = 0.8, Cox proportional hazard model: HR: 1.22, 95% CI: 0.62–2.41, *P* = 0.56. Shaded regions indicate the 95% confidence intervals (the first GDI group: thin shaded region, the second GDI group: thick shaded region). horizontal axis: months

When the eyes with combined TRAB/GDIS were excluded, the survival-curve rate at 3-, 5-, and 10-years postoperative as evaluated by Criteria 1 (Fig. 4) in the first GDI group was 73.8%, 56.3%, and 52.4%, respectively, while that at 3- and 5-years postoperative in the second GDI group was 77.1% and 54.5%, respectively. There was no statistically significant difference in the survival-curve rates between the first GDI group and the second GDI group (*P* = 0.2). The survival-curve rates corrected by patient background with the Cox proportional hazard model also showed the same results (HR: 0.81, 95% CI: 0.46–1.44, *P* = 0.48). On the other hand, the Kaplan-Meier survival-curve rate defined with Criteria 2 (Fig. 5) at 3-, 5-, and 10-years postoperative in the first GDI group was 37.3%, 29.0%, and 19.2%, respectively, and that at 3- and 5-years postoperative in the second GDI group was 42.1% and 10.2%, respectively. There was no statistically significant difference in the survival-curve rates between the first GDI group and the second GDI group (*P* = 0.80). The survival rates corrected by patient background with the Cox proportional hazard model also showed the same results (HR: 1.22, 95% CI: 0.62–2.41, *P* = 0.56).

There was no serious early postoperative complication affecting the outcome of GDIS. The complications that did occur post GDIS are listed in Table 3. No significant difference in tube exposure, hypotension, macular edema, endophthalmitis, persistent corneal edema, diplopia, or other complications was found between the first GDI group and the second GDI group. However, tube exposure occurred in 19 eyes (in 14 of the first GDI group eyes and in 5 of the second GDI group eyes). Of those 19 eyes, the tube was exposed within a few months postoperative in 4 eyes, and the mean period post surgery in which the tube was exposed in the remaining 15 eyes was 77.3 ± 44.1 months. Endophthalmitis occurred in 1 eye after tube exposure resulted in blindness. The mean elapsed time between GDIS and tube exposure was 34.3 months. In addition, there were 4 eyes of 5 cases in which tube exposure occurred post implantation of the second GDI, but not post implantation of the first GDI.

**Table 3:** Complications: the first GDI group vs. the second GDI group

	the first GDI group	the second GDI group	*P*-value
No. of eyes	136	32	
Tube exposure〈n, (%)〉	14 (10.3%)	5 (15.6%)	0.60
Hypotension / shallow anterior chamber〈n, (%)〉	6 (4.4%)	1 (3.1%)	1.00
Macular edema〈n, (%)〉	3 (2.2%)	0	1.00
Endophthalmitis〈n, (%)〉	0	1 (3.1%)	0.19
Bullous keratopathy / corneal decompensation 〈n, (%)〉	6 (4.4%)	4 (12.5%)	0.10
Loss of light perception〈n, (%)〉	1 (0.7%)	0	1.00
Other〈n, (%)〉	21 (15.4%)	7 (21.9%)	0.57
Total〈n, (%)〉	46 (33.8%)	17 (53.1%)	0.08

Fisher’s exact test / chi-square test

Prior to surgery, the mean CEC density in the second GDI group (i.e., 2102.6 ± 647.7 cells/mm^2^) was significantly lower (*P* < 0.01) than that in the first GDI Group (i.e., 2423.3 ± 579.0 cells/mm^2^), while that at the final follow-up visit in the second GDI group (i.e., 1422.4 ± 688.8 cells/mm^2^) was also found to be significantly lower (*P* < 0.01) when compared with that in Group 1 (i.e., 1995.3 ± 706.4 cells/mm^2^). Moreover, the mean speed of CEC density reduction in the second GDI group (i.e., -208.5 ± 175.9 cells/mm^2^ per year) was significantly faster (*P* < 0.01) than that in the first GDI group (i.e., -95.7 ± 175.9 cells/mm^2^ per year), and it was also found to be significantly higher via multivariate analysis adjusted for previous glaucoma or cataract surgeries performed (*P* < 0.05). As for the annual speed of BCVA loss (*P* = 0.92) and VF deterioration (*P* = 0.93) post surgery, no statistically significant differences were found between the two groups.

Young patient age at the time of surgery was the only significant risk factor among the patient background parameters (i.e., 1–14) in the eyes of the first GDI group (odds ratio [OR]: 0.95, 95% Cl: 0.91-1.00, *P* = 0.03) and in the eyes of the second GDI group (OR: 0.79, 95% Cl: 0.61–1.01, *P* = 0.06).

## Discussion

In most previous studies on GDIS, a target IOP of ≤ 21 mmHg has widely been applied. However, in patients with advanced-stage VFD, an IOP of 20 mmHg or high teens post GDIS may not be safe. In the Early Manifest Glaucoma Trial (EMGT) [10], a randomized controlled trial of POAG patients, Bengtsson et al. reported that the mean postoperative IOP was 16.5 mmHg in the non-progressive VFD group. In the Advanced Glaucoma Intervention Study (AGIS) [11], the investigators reported that the mean postoperative IOP was 12.3 mmHg, and that there was no worsening of VF. Moreover, it was reported that in Japanese POAG patients with an IOP of < 12 mmHg, a VF worsening of < -0.3dB per year was not observed [12]. Thus, in this present study, we set another criteria of an IOP of ≤ 14 mmHg as Criteria 2, which may be appropriate for patients with severe VF deterioration.

Although our study was retrospective, the results in our the first GDI group (i.e., the first GDIS) were found to be slightly worse when compared to the findings in previous prospective studies [13, 14] (Table 4), possibly due to the fact that the mean age of the patients in this study (i.e., 61.0 ± 11.2 years) was lower than that in the above-cited prospective studies (i.e., 64 ± 14 years and 70.9 ± 11.0 years, respectively). That finding suggests that in our patients, the onset of POAG occurred at a younger age in comparison to the patients in those previous prospective studies. Our findings revealed that a young age at the time of GDIS was the only significant risk factor among the various patient background parameters. It should be noted that if a more severe criteria had been set for postoperative IOP, e.g., ≤ 14 mmHg, then the success rates at 5-years postoperative in all eyes would have reduced from 60.4% (Criteria 1: ≤21 mmHg) to 31.5% (Criteria 2: ≤14 mmHg). Those findings indicate that when GDIS is performed in POAG patients in whom a postoperative IOP of ≤ 14 mmHg is required, it may be difficult to achieve for a long-term period post surgery.

**Table 4:** Comparison of the results of the first GDIS for refractory POAG with the findings in the previous reports

GDI type	Gedde et al. Am J Ophthalmol 2012	Christakis et al. Am J Ophthalmol 2017	This study
No. of eyes	107	247	136
POAG (%)	82%	49%	100%
	BGI	Only BGI excerpts	BGI DP-Molteno Implant
Postoperative IOP (mmHg)	14.4	13.2	15.5
Postoperative Glaucoma medication	1.4	1.5	2.5
Success rate after 5 years	5<IOP ≦ 21mmHg 70.2%	6≦IOP ≦ 21mmHg 65%	5<IOP ≦ 21mmHg 56–60%
	5<IOP ≦ 17mmHg 68.2%	6≦IOP ≦ 18mmHg 63%	
	5<IOP ≦ 14mmHg 47.7%	6≦IOP ≦ 15mmHg 52%	5<IOP ≦ 14mmHg 29–32%

BGI, Baerveldt glaucoma implant; DP-Molteno Implant, double-plate Molteno implant; GDI, glaucoma drainage implant; IOP, intraocular pressure; POAG, primary open-angle glaucoma

It has been reported in previous studies that the mean postoperative IOP and medication score in cases that undergo the second GDIS is 13.4–18.5 mmHg and 1.2–2.8, respectively [15–20]. In this present study, the mean postoperative IOP and medication score was 14.8 ± 5.4 mmHg and 3.1 ± 1.6, respectively, which is similar to the findings in those previous reports. In addition, the 5-year-postoperative survival rate with Criteria 1 in our the second GDI group patients was 54.5%, which is also similar to the findings in those previous reports [15–20]. However, if the Criteria 2 is set, the survival rate at 5-years postoperative reduces to 25.6%. Thus, and as stated above, it is difficult to achieve a target IOP post surgery in eyes that require an IOP in the low teens.

To the best of our knowledge, this is the first comparative study between the first and second GDIS using a non-valved GDI performed by a single surgeon for cases of POAG. In a recent report by Yoon and Vajaranant [21], in which the authors performed a meta-analysis of the findings in 9 previous studies, it was found that the second GDIS tended to fail earlier. That study simply compared findings with those in the tube versus trabeculectomy (TVT) study [14], yet without the use of statistical methods. The ratio of POAG patients in those studies [21] varied between 25 and 47.4%. In this present study involving only POAG patients, no significant difference was found between the patients who underwent the first GDIS (the first GDI group) and those who underwent the second GDIS (the second GDI group) in regard to IOP reduction, the number of glaucoma medications used, and success rate at 5-years postoperative. If the eyes that underwent combined TRAB/GDI were excluded, there was no difference of postoperative success rate. In addition, there was no significant difference in BCVA, VFD change, and surgical complications between the two groups. Those results indicate that the second GDIS may be equally effective with the first GDIS in POAG patients.

The success of GDIS may be greatly influenced by the age of the patient, the type of glaucoma, and the patient-specific fibrotic reaction around the plate. In this present study, which was limited to POAG patients, a younger patient age was the statistically significant risk factor for failure in the first GDI group (*P* = 0.03), and that same tendency was found in the second GDI group (*P* = 0.06). In other studies, it has been reported that the number of intraocular surgeries [22], a previous TRAB [17], and younger age [19] are risk factors for failure in patients who undergo GDIS. However, the percentage of POAG patients included in the study ranged between 18 and 54% [17, 19, 22]. The fibrosis around the plate may occur more intensely in younger-age patients or in eyes that have undergone a previous intraocular surgery. However, the onset of POAG occurring at a younger age may have more greatly impaired the outflow pathway, which may have resulted in the need for multiple glaucoma surgeries. In a previous study in which we compared Schlemm’s canal (SC) and trabecular meshwork (TM) morphologies between JOAG and POAG, our findings revealed developmental abnormalities in JOAG in addition to age-related abnormalities in the TM and SC [23].

In this study, complications, other than tube exposure, were fewer in comparison to those reported in previous studies (Table 5) [14, 16, 18–21, 24, 25]. Tube exposure may be one of the most serious complications encountered in GDIS, as endophthalmitis can occur if the initiation of treatment is delayed. In this present study, tube exposure occurred in 19 eyes, and the mean elapsed time between GDIS and tube exposure was 34.3 months. The early tube exposure that occurred within a few months in 4 of those 19 eyes may have been due to insufficient conjunctival covering over the patch at the time of GDIS. In the other 15 eyes, the exposure took much longer to occur post surgery when compared to the findings reported in previous studies. Thus, excluding the eyes in which early tube exposure occurred in this study, the reason for the higher incidence of tube exposure in our patients (mean period post GDIS: 77.3 months) may possibly be due to the longer follow-up period post surgery. Reportedly, some possible reasons for tube exposure include a lower site of insertion [26], a younger patient age [27], and multiple surgeries [28]. We experienced 4 eyes in which the second GDI became exposed despite the fact that there was no thinning of the scleral patch in the first GDI. Thus, a match between the donor sclera and recipient may also be important. Warning the patient of possible redness on the limbus at the insertion site of the tube may also be important if the patch becomes thin or melted.

**Table 5:** Comparison with previously reported complications

	The first GDI surgery			The second GDI surgery						
	Gedde et al. 2012	Budenz et al. 2016	**This** **Study**	Yoon et al. 2020	Posarelli et al. 2020	Fatehi et al. 2018	Hu et al. 2016	Anand et al. 2010	Jimenez et al. 2016	**This Study**
Tube exposure (%)	4.6	4.5	10.3	5.6	4		6.1	0	1.7	15.6
Hypotony / shallow anterior chamber (%)	11	4.5	4.4		4		1.5	4.7	5.2	3.1
Macular edema (%)	5	7.2	2.2				3.1		3.4	0
Endophthalmitis (%)	1	2.2	0	2.0	4	1.8		0	1.7	3.1
Bullous keratopathy / corneal decompensation (%)	16	20.4	4.4	9.5	29		14	16.3	17.2	12.5

Several previous studies have reported on a loss of CEC density post GDIS, which is thought caused by physical contact of the tube to the cornea at its insertion point [29], the distance of the tube tip to the cornea [30], a change in the location of the tube during the postoperative time course [31], a foreign body reaction against the tube material [32], or turbulence at the tip of the silicon tube [33]. Kim et al. [34] reported that post AGV implantation, the mean loss of CEC density per month was − 17.5 cells/mm^2^ (0.84%), while that reported by Zhang et al. [35] was − 29.3 cells/mm^2^ (1.37%). Iwasaki et al. reported that in Japanese patients who underwent implantation of the BGI, the loss of CEC density per year was 203 cells/mm^2^ (12.1%) [36]. In our the first GDI group patients, the mean speed of CEC reduction was rather slow (i.e., -95.7 cells/mm^2^ per year) when compared to that reported in other studies. In Japan, the loss of CEC density in the early period post GDIS was first pointed out by Chihara et al. in 1992 [37]. Thus, in our hospital, great care is taken at the time of GDI insertion to run the tube close and parallel to the iris, and penetrate into the TM not beyond Schwalbe’s line, thus resulting in the tube position being far from the cornea [38, 39]. In this study, in the eyes of the first GDI group and the second GDI group, CEC density was reduced at a speed of -95.7 cells/mm^2^/year and − 208.5 cells/mm^2^/year, respectively. The significantly higher speed of CEC density loss observed in the eyes implanted with the second GDI (*P* < 0.01) may have been the result of the CEC loss associated with the first GDI or with previous glaucoma surgeries.

There has been a discussion as to whether the second GDIS or excision of the encapsulated bleb [40, 41] should be performed if the first GDIS fails. In a comparison study, the second GDIS was found to offer better IOP control than the excision of an encapsulated bleb [41]. Trans-scleral cyclophotocoagulation (TSCPC) [42–44] and endoscopic cyclophotocoagulation (ECP) [45–47] are other surgical options that can be applied in eyes with a failed GDIS. Reportedly, TSCPC has a large IOP lowering effect, and provides the same success rate with no significant complications when compared to the second GDIS [43]. On the other hand, and even though the second GDIS results in a longer survival rate, there are more side effects when compared to TSCPC [44]. Reportedly, ECP results in the same IOP reduction and success rate as that associated with the second GDIS [46] and has less complications compared to TSCPC [47]. Destruction of the ciliary body is thought to be the final surgical option in cases of failed glaucoma surgery due to unexpected hypotension, VF loss, or phthisis [48]. Thus, further study is needed to fully elucidate the best treatment option for cases of a failed the second GDIS.

It should be noted that this present study did have limitations. First, this study was a retrospective study. Second, the background of the patients may have confounded the results, as the proportion of males and the number of previous TRAB surgeries performed in the second GDIS group was higher than that in the first GDIS group.

In conclusion, both the first GDIS (the first GDI group) and second GDIS (the second GDI group) were found effective for lowering IOP in cases afflicted with POAG, and the survival rates at 5-years postoperative in the first GDI group and the second GDI group were 60.4% and 61.2%, respectively. However, if the criteria for success is strictly set as a postoperative IOP in the low teens, the survival rates at 5-years postoperative in the first GDI group and the second GDI group decreases to 31.7% and 25.6%, respectively. Thus, both the first and second GDIS may be insufficient for the treatment of POAG patients in whom the postoperative IOP must be in the low teens. Moreover, younger-age POAG patients are the only significant risk factor among the patient background parameters.

## Data Availability

The datasets used and/or analyzed during the current study are available from the corresponding author on reasonable request.

## List of abbreviations

GDIS: glaucoma drainage implant surgery
IOP: intraocular pressure
GDI: glaucoma drainage implant
POAG: primary open-angle glaucoma
BGI: Baerveldt glaucoma implants
VFD: visual field defect
VF: visual field
TRAB: Trabeculectomy
PPV: pars plana vitrectomy
TLO: trabeculotomy
BCVA: best-corrected visual acuity
CEC: corneal endothelial cell
SD: standard deviation
HR: hazard ratio
CI: confidence interval
EMGT: the Early Manifest Glaucoma Trial
AGIS: the Advanced Glaucoma Intervention Study
double-plate Molteno implant: DP-Molteno implant
TVT: tube versus trabeculectomy
SC: Schlemm’s canal
TM: trabecular meshwork
JOAG: juvenile open-angle glaucoma
TSCPC: Trans-scleral cyclophotocoagulation
ECP: endoscopic cyclophotocoagulation
